# Intermuscular Fat and Physical Activity Levels Relative to Exercise Capacity Change During Breast Cancer Treatment

**DOI:** 10.1016/j.jaccao.2025.01.009

**Published:** 2025-02-25

**Authors:** Lauren Daniel, Moriah P. Bellissimo, Ralph B. D’Agostino, Kristine C. Olson, Amy C. Ladd, Kerryn W. Reding, Kathryn E. Weaver, Glenn J. Lesser, Bonnie Ky, W. Gregory Hundley, UPBEAT Study Team

**Affiliations:** a VCU Pauley Heart Center, Division of Cardiology at Virginia Commonwealth University, Richmond, Virginia, USA; b Department of Biostatistics and Data Science, Wake Forest School of Medicine, Winston-Salem, North Carolina, USA; c School of Nursing, University of Washington, Seattle, Washington, USA; d Department of Social Sciences and Health Policy, Wake Forest School of Medicine, Winston-Salem, North Carolina, USA; e Section on Hematology and Oncology, Department of Internal Medicine, Wake Forest School of Medicine, Winston-Salem, North Carolina, USA; f Department of Medicine, Perelman School of Medicine at the University of Pennsylvania, Philadelphia, Pennsylvania, USA

**Keywords:** anthracycline, breast cancer, cardio-oncology, exercise capacity, imaging, intermuscular fat, physical activity, treatment

Excessive intermuscular fat (IMF) is associated with decreases in exercise capacity (EC) in breast cancer (BC) survivors who previously received potentially cardiotoxic chemotherapy.^1^ Insufficient physical activity (PA) is associated with accumulation of IMF in participants without cancer and with decreased 6-minute walk distance (6MWD) in cancer survivors receiving potentially cardiotoxic chemotherapy.^2^^,^^3^ In this study we investigated the relationship among PA levels, IMF, and EC change in BC survivors receiving potentially cardiotoxic chemotherapy.

This was a secondary analysis of NCT02791581, a multicenter, prospective cohort study of women diagnosed with stage I to III BC conducted through the Wake Forest National Cancer Institute Community Oncology Research Program Research Base (grant UG1CA189824) and the Eastern Cooperative Oncology Group–American College of Radiology Imaging Network Cancer Research Group (grant UG1CA189828) under a National Cancer Institute Central Institutional Review Board–approved and local site–approved protocol. All patients provided written informed consent.

BC survivors enrolled in this study were women ≥18 years of age scheduled to receive potentially cardiotoxic chemotherapy for stage I to III BC (n = 128) and 76 family or friend comparators without cancer. BC patients completed magnetic resonance imaging, the 6-minute walk test, and the Godin-Shephard leisure-time PA questionnaires before and 3 months after chemotherapy initiation. Comparators underwent the same testing separated by 3 months. Participants were categorized as highly or less active according to baseline Godin-Shephard leisure-time PA questionnaire (which measures weekly exercise frequency). This questionnaire determined placement into the less (moderately or insufficiently active) or highly active groups. All participants underwent magnetic resonance imaging in the axial plane at the level of second lumbar vertebra, and IMF of the paraspinal muscles was determined using sliceOmatic software (TomoVision) from a manual paired, blinded read of the images from the 2 time points. Participants were grouped by the unstandardized baseline ratio of IMF to skeletal muscle (SM) into groups above and below the pooled median value.

The primary outcome was the 3-month change in 6MWD. An analysis of covariance model was used to examine the relationship among PA, IMF, and EC while adjusting for baseline 6MWD, PA, and IMF, as well as age, race, smoking history, hematocrit, and serum creatinine. SAS version 9.4 (SAS Institute) was used for analysis. Demographic characteristics between the cancer and comparator groups were compared using Student’s *t*-test or the Wilcoxon rank sum (nonparametric) test (for continuous variables) or the chi-square tests (for categorical variables).

Mean age (53.3 ± 12.5 years), race (76% White, 16% Black, 8% other), and median body mass index (28.4 kg/m^2^; Q1-Q3: 23.5-33.8 kg/m^2^) were similar between cancer survivors and comparators. Sixty percent were postmenopausal. These demographic assessments were similar to previously published studies of women receiving treatment for BC.^1^^,^^3^ All patients received potentially cardiotoxic chemotherapy, with 57% receiving anthracycline chemotherapy. Of the 128 cancer patients, 26 received trastuzumab, 82 received cyclophosphamide, 18 received tamoxifen, 42 received anastrozole, and 74 received doxorubicin (mean dose 224.8 mg/m^2^). BC survivors demonstrated an average ± SE baseline 6MWD of 464.7 ± 7.5 m. BC survivors receiving trastuzumab had a greater decrease in 6MWD (mean ± SE, −57.8 ± 8.2 m) compared with those who did not receive trastuzumab (mean ± SE, −18.8 ± 17.6 m; *P* = 0.034). Use of anastrozole (*P* = 0.086), tamoxifen (*P* = 0.095), cyclophosphamide (*P* = 0.95), and doxorubicin (*P* = 0.10) were not associated with changes in 6MWD. Two participants received tamoxifen, and 1 received anastrozole. BC survivors experienced declines in 6MWD over 3 months relative to comparators (mean ± SE, −25.1 ± 6.9 m and 9.8 ± 9.9 m, respectively; *P* < 0.001).

Relative to the median value (0.34), 80% of cancer survivors and 29% of comparators displayed high IMF/SM ratios (*P* < 0.001). The changes in 6MWD were not associated with race, age, hematocrit, serum creatinine, tobacco use, cardiovascular risk factors, baseline ejection fraction, change in ejection fraction from baseline to 3 months, history of myocardial infarction, or use of beta-blockers, angiotensin-converting enzyme inhibitors, diuretic agents, or lipid-modifying agents. In BC survivors and comparators, highly active individuals with low baseline IMF walked further after 3 months relative to those with physical inactivity and/or high IMF (Table 1). All less active groups experienced declines in 6MWD, regardless of muscle quality. This observation raises the possibility that low IMF/SM ratio does not compensate for insufficient PA when measuring changes in 6MWD during chemotherapy. Physical inactivity during 3 months of BC treatment may be related to a persistent elevation of IMF or a reduction in SM oxygen extraction from surrounding vasculature during exercise.^4^ These hypotheses require further study.

**Table 1:** Descriptive Data by Participant Group for 6MWD, Physical Activity, and Muscle Quality

	Patients	3-Month 6MWD, m	Δ6MWD, m	Age, y	Postmenopausal	IMF/SM Ratio	Muscle Surface Area, cm^2^/m^2^	Anthracyclines, % of Total	Beta-Blockers	ACE Inhibitors	Diuretic Agents	Lipid-Modifying Agents
Comparator, IMF ↑,^a^ highly active	8 (4)	466.9 (440.4–493.4)	3.2 (−51.9 to 58.3)	60.4 ± 5.7	8 (100)	0.38 ± 0.04	34.6 ± 5.0	0	1	0	2	2
Comparator, IMF ↑,^a^ less active	14 (7)	474.4 (453.8–494.9)	−5.4 (−43.3 to 32.4)	56.0 ± 8.9	11 (79)	0.41 ± 0.05	34.4 ± 3.8	0	6	1	2	4
Comparator, IMF ↓,^b^ highly active	28, (14)	497.7 (482.3–513.2)	49.7 (20.2 to 79.3)	47.3 ± 12.9	10 (35)	0.28 ± 0.14	36.3 ± 6.1	0	0	2	1	3
Comparator, IMF ↓,^b^ less active	26 (13)	469.8 (453.9–485.6)	−9.1 (−39.4 to 21.2)	43.8 ± 13.8	7 (27)	0.30 ± 0.15	37.3 ± 7.4	0	0	1	2	3
Cancer, IMF ↑,^a^ highly active	32 (16)	460.3 (445.5–475.1)	−20.3 (−45.5 to 4.9)	57.8 ± 9.8	23 (72)	0.43 ± 0.08	41.4 ± 7.4	25	2	2	6	6
Cancer, IMF ↑,^a^ less active	48 (23)	452.5 (441.7–463.2)	−43.6 (−65.2 to −21.9)	59.1 ± 11.4	11 (39)	0.43 ± 0.07	38.7 ± 7.1	38	6	7	14	21
Cancer, IMF ↓,^b^ highly active	15 (7)	476.9 (455.7–498.1)	9.2 (−27.6 to 46.1)	51.3 ± 10.9	10 (67)	0.29 ± 0.14	42.2 ± 8.6	12	0	0	1	1
Cancer, IMF ↓,^b^ less active	33 (16)	451.9 (439.2–464.6)	−43.3 (−68.3 to −18.3)	52.7 ± 11.6	17 (52)	0.29 ± 0.12	38.4 ± 6.1	25	3	3	4	9

Values are n (%), mean (95% CI), mean ± SD, or n, unless otherwise indicated. 3-month 6MWD and Δ6MWD were adjusted for age. Muscle surface area was indexed to body surface area. “Highly active” participants met U.S. physical activity guidelines, and “less active” patients did not.

6MWD = 6-minute walk distance; ACE = angiotensin-converting enzyme; IMF = intermuscular fat; SM = skeletal muscle.

a Baseline IMF/SM ratio greater than median.

b Baseline IMF/SM ratio less than median.

In conclusion, among BC survivors receiving 3 months of potentially cardiotoxic chemotherapy, sufficient PA and low IMF/SM ratio were associated with maintained 6MWD, whereas insufficient PA and/or high IMF/SM ratio were associated with decreased 6MWD. This study motivates future research into the mechanisms involved in the relationship among IMF, activity, and BC treatment–associated exercise intolerance and whether interventions that increase activity or improve muscle quality attenuate BC treatment associated declines in EC. Limitations of our study include a small sample size, self-reported PA, observational study design, and use of 6MWD, which could be limited by factors inhibiting mobility.

## Funding Support and Author Disclosures

This research was supported in part by Virginia Commonwealth University and the Wake Forest School of Medicine (National Institutes of Health grant R01CA199167), the Wake Forest National Cancer Institute Community Oncology Research Program Research Base (National Institutes of Health grant 2UG1CA189824), and the Eastern Cooperative Oncology Group–American College of Radiology Imaging Network National Cancer Institute Community Oncology Research Program Research Base (grant UG1CA189828). The authors have reported that they have no relationships relevant to the contents of this paper to disclose.
